# Contrasting effect of irrigation practices on the cotton rhizosphere microbiota and soil functionality in fields

**DOI:** 10.3389/fpls.2022.973919

**Published:** 2022-10-18

**Authors:** Bin Peng, Shuai Zhao, Samiran Banerjee, Wenxuan Mai, Changyan Tian

**Affiliations:** ^1^ State Key Laboratory of Desert and Oasis Ecology, Xinjiang Institute of Ecology and Geography, Chinese Academy of Sciences, Urumqi, China; ^2^ University of Chinese Academy of Sciences, Beijing, China; ^3^ Department of Microbiological Sciences, North Dakota State University, Fargo, ND, United States

**Keywords:** cotton, drip irrigation, rhizosphere, bacterial community, functional potential

## Abstract

Drip irrigation under plastic film mulch is a common agricultural practice used to conserve water. However, compared to traditional flood irrigation with film mulch, this practice limit cotton root development from early flowering stage and may cause premature senescence in cotton. Changes of root will consequently shape the composition and activity of rhizosphere microbial communities, however, the effect of this farming practice on cotton rhizosphere microbiota remains poorly understood. This study investigated rhizosphere bacteria and soil functionality in response to different irrigation practices —including how changes in rhizosphere bacterial diversity alter soil nutrient cycling. Drip irrigation under plastic film mulch was shown to enhance bacterial diversity by lowering the salinity and increasing the soil moisture. However, the reduced root biomass and soluble sugar content of roots decreased potential copiotrophic taxa, such as *Bacteroidetes*, *Firmicutes*, and Gamma-*proteobacteria*, and increased potential oligotrophic taxa, such as *Actinobacteria*, *Acidobacteria*, and *Armatimonadetes*. A core network module was strongly correlated with the functional potential of soil. This module not only contained most of the keystone taxa but also comprised taxa belonging to *Planctomycetaceae*, *Gemmatimonadaceae*, *Nitrosomonadaceae*, and *Rhodospirillaceae* that were positively associated with functional genes involved in nutrient cycling. Drip irrigation significantly decreased the richness of the core module and reduced the functional potential of soil in the rhizosphere. Overall, this study provides evidence that drip irrigation under plastic film mulch alters the core bacterial network module and suppresses soil nutrient cycling.

## Introduction

Cotton (*Gossypium hirsutum* L.) is an important crop in both the agriculture and textile industry (Constable and Bange, 2015). Xinjiang is the largest cotton-producing region in China with a cotton planting area of approximately 2.5 million hectares (ha) (NBSC, 2020). As a result of low temperatures that occur during the seedling stage and water shortages, the combination of drip irrigation and plastic film mulch is extensively used to increase topsoil temperature and save water in dryland farming areas (Zong et al., 2021). Plastic film mulching is applied nearly in all cotton fields in Xinjiang; and cotton fields that use drip irrigation under plastic film mulch currently comprise a nearly 1.2 million ha area (NBSC, 2020). However, compared to traditional flood irrigation with film mulch, this practice reduces root biomass and length (from early flowering stage) and often causes premature senescence in cotton (Mai et al., 2014). In addition, drip irrigation alters the nutrient content in rhizosphere soils (Zhu et al., 2013). This is particularly important because changes in root traits and rhizosphere conditions can affect rhizosphere microbes that are vital for plant growth and fitness (Berendsen et al., 2012; Zhang et al., 2017). While previous studies have explored the effects of drip irrigation on cotton yield, less attention had been paid to its influence on soil biological properties, especially on the rhizosphere microbial community. Thus, knowledge of how rhizosphere soil conditions under drip irrigation alter the rhizosphere microbiome and affect microbially-driven soil functions remains limited.

Rhizosphere microbes determine plant nutrient utilization by actively participating in carbon or nitrogen fixing and releasing available micronutrients. As a result, changes in rhizosphere microbes can influence the development of plants (Zhang et al., 2017). In practice, drip irrigation under plastic film mulch limits primary root development (Mai et al., 2014), and enriches ${\text{NO}}_{3}^{-}$ -N in rhizosphere soils (Zhu et al., 2013), potentially impacting the structure and function of rhizosphere microbes. For example, variations in root biomass and length influence the rhizosphere priming effect and the subsequent release of labile organic substances (Zhang et al., 2017). Labile organic substances promote the growth of microbial r-strategist groups such as Beta*-proteobacteria*, Gamma-*proteobacteria*, *Firmicutes*, *Gemmatimonadetes*, and *Bacteroidetes* (Zhou et al., 2018) and are linked to the decomposition of soil organic matter and solubilization of insoluble minerals by rhizosphere microbes (Zhang et al., 2017). Soil available N also affects *cbbL*-carrying autotrophic bacterial composition (Zhou et al., 2019), and N fertilizers suppress various diazotrophic taxa and N fixation rates (Fan et al., 2019). It should be noted that drip irrigation under plastic film mulch increases the amount of residual membrane, which can impose selective pressure on distinct rhizosphere bacterial taxa (Qi et al., 2020). Thus, drip irrigation under plastic film mulch can induce complex rhizosphere conditions and change the characteristics of the rhizosphere microbiome.

Complex potential associations among thousands of soil organisms can be visualized as networks within the microbial community. Networks reveal the factors driving microbial associations in response to environmental disturbance, and provide critical information on microbial taxa associated with soil functioning (Chaffron et al., 2010; Banerjee et al., 2018). For example, it has been found wheat rhizosphere fungal network modules composed of saprotrophs or pathogens; and the modules are affected by tillage (Li et al., 2021). Network analysis studies also have identified several keystone taxa that are critical to microbial community functioning (Banerjee et al., 2021; Zheng et al., 2021). One recent study found that taxa within the core module were positively associated with functional genes involved in maintaining soil nutrient cycling (Fan et al., 2021). Thus, it is worth investigating whether keystone species are also harbored within the core module. Manipulating the rhizosphere microbes can favor plants for sustainable agricultural gains (Zhang et al., 2017) so characterizing the key players involved in nutrient cycling is essential to plant production and supports soil functioning in a more managed ecosystem (Banerjee et al., 2018; Fan et al., 2021). This is especially important after the combined use of plastic film mulch and drip irrigation, which can alter plant traits and soil nutrient availability.

The current study uses three irrigation treatments (flooding irrigation with single plastic film mulch and drip irrigation under single or double plastic film mulch) during a 2-year field experiment in an arid region of China. Shifts in the bacterial community, functional genes, RubisCO activity, and potential rates of nitrification and denitrification were assessed to characterize the bacterial response to drip irrigation-induced effects on rhizosphere nutrient cycling. There were two main objectives: (i) to compare microbial responses to the three different irrigation practices and (ii) to evaluate the effect of irrigation practices on rhizosphere C and N cycling processes. It was hypothesized that keystone taxa in the core module are linked to soil nutrient cycling processes, and drip irrigation under plastic film mulch affects soil functional potential by influencing the core network module.

## Materials and methods

### Experimental design and sampling

A 2-year field experiment was initially established in 2019 at YuShuGou in the Xinjiang Uyghur Autonomous Region of China (44°09′59″N, 87°59′56″E). This area has a typical temperate continental climate with an average annual precipitation of 190 mm and an average annual temperature of 6.8^°^C. The experiment consisted of three treatments: flooding irrigation with film mulch (FSM), drip irrigation with single film mulch (DSM), and drip irrigation with double film mulch (DDM) (**Figure S1**). Each treatment had six replicates (8 m × 20 m per plot for each replicate) arranged in a randomized block design, equaling 18 plots in total with guard rows (width of 1 m) between each plot. Nitrogen fertilizer and nitrogen fertilizer with water applications were managed as described by Mai et al. (2014). In brief, 4000 m^3^ water/hm^2^ and 340 kg urea/hm^2^ were applied each year for drip irrigation with mulch film, and 6000 m^3^ water/hm^2^ and 400 kg urea/hm^2^ were used each year for flooding irrigation with mulch film. The rates and dates of water and fertilizer application were consistent with local cotton agronomic practices (**Table S1**).

Plants and soil were sampled on 20 August 2020 at the fruiting stage of the cotton (about 18 weeks after sowing) and rhizosphere soil samples were collected as described previously (Mo et al., 2019). To ensure that the sample size of rhizosphere soil was similar and comparable across treatments, roots were collected within a 20-cm soil layer. Loose soil was obtained to filter out film residue. During sample collection, the rhizosphere soil was carefully scraped off the root surface using a sterile scalpel. The samples were then transported on ice to the laboratory for analysis. Some of the soil was stored at -80°C for DNA extraction and the remainder were stored at 4°C for chemical analyses.

### Plant traits and soil physicochemical properties

For each treatment, 120 plant samples (20 for each replicate) were dried to a constant weight to determine the biomass. Some of the cotton root samples (10 for each plot) were also used to measure biochemical traits (Wang and Huang, 2015). Phenol content was estimated using the Folin–Ciocalteu assay, soluble sugar content was assessed using Anthrone colorimetry, free amino acids were measured using Ninhydrin colorimetry, and the absorbance was determined using an ultraviolet (UV) spectrophotometer (Cary 60, Agilent Technologies, USA). Soil physicochemical properties, including soil soluble salt content, pH, soil organic matter, nitrate-nitrogen, and ammonium nitrogen, were measured as previously described (Zhao et al., 2019; Zhao et al., 2020). Water-extractable organic carbon (WEOC) was determined as outlined by Zhu et al. (2020).

### Analyses of the soil carbon-fixation, nitrification, and denitrification potentials

RubisCO activity was determined as described by Wu et al. (2015). In brief, a soil solution was mixed with the reaction system, and a preparation lacking RuBP was used as a blank. Ribulose bisphosphate (50 μL, 25 mM) was added to the mixture for 30 s, the absorbance was measured at 340 nm, and the RubisCO activity (nmol CO_2_ min ^-1^ per kg dry soil) was calculated. Soil potential nitrification (PNR) was assessed according to a method described by Stark and Firestone (1995). In brief, field-moist soil (10 g) was added to a 250 ml Erlenmeyer flask with 100 ml nitrification solution consisting of KH_2_PO_4_ (280 mM), K_2_HPO_4_ (720 mM), and (NH_4_)_2_SO_4_ (500 mM). The samples were continuously shaken at 110 rpm and 10 ml of each sample solution was collected at 24, 48, 72, 96, 130, and 154 hours, filtered, and immediately stored at -20° C until analysis. NH^4+^-N and ${\text{NO}}_{3}^{-}$ -N concentrations were then determined. The denitrification potential (DEA) was tested as described by Griffiths et al. (1998). In brief, 10 g of soil was weighed into a glass container that received 10 ml of a solution containing 1 mM KNO_3_ and 1 mM glucose. High purity (99.99%) helium was flushed into the container to create anaerobic conditions under atmospheric pressure. For DEA samples, 10 ml of gas was taken out and replaced by injecting 10 ml of acetylene. The slurries were shaken at 100 rpm for 154 h and the gas samples were analyzed. DEA rates were determined for each sample by calculating the slope of the linear regression model at four time points.

### DNA extraction and quantitative PCR

Total soil DNA from each sample was extracted using the E.Z.N.A. Soil DNA kit (Omega, USA) and diluted in TE buffer (10 mM Tris-HCl, 1 mM EDTA, pH 7.0). The abundance of each functional gene, *cbbL*, *β-glu, ChiA, nifH*, AOB*-amoA*, *narG*, *nirK*, and *cnorB* was determined by quantitative PCR (qPCR) using the LightCycler^®^ 480 system (Roche Applied Science). A 20 μL reaction contained 10 μL SYBR Premix Ex Taq™ (Takara, Dalian, China), 0.4 μL of each forward and reverse primer, and 2 μL of template DNA (10 ng μL^-1^). Standard curves showed high correlation coefficients (R^2^ >0.98), and the PCR efficiency was at least 85% in all cases. Details regarding the primers, thermal cycling conditions, and quality assessment are provided in **Table S2**.

### High-throughput sequencing

The hypervariable V4-V5 region of the bacterial 16S rRNA gene was amplified using 515F (5’-GTGCCAGCMGCCGCGG-3’) and 907R (5’-CCGTCAATTCMTTTRAGTTT-3’) primers (Yusoff et al., 2013). The 25 μL PCR reactions contained 1 μL of each primer (10 μM), 2 μL DNA (10 ng μL^−1^), and 22 μL Platinum PCR SuperMix (Invitrogen, Shanghai, China). The thermal program was as follows: 95°C for 3 min, followed by 27 cycles at 95°C for 30 s, 50°C for 45 s, and 72°C for 1 min with a final extension at 72°C for 10 min. Sequencing was performed on the Illumina MiSeq platform at Majorbio BioPharm Technology Co., Ltd. (Shanghai, China), and the raw sequences were deposited in GenBank under the BioProject PRJNA793144. Using QIIME 1.9.0, the raw data was processed and trimmed (http://qiime.org; Caporaso et al., 2010). Low-quality reads (length<200 bp and mean quality score<30) were removed, while reads that overlapped by >10 bp were assembled. Chimeras were removed using the Uchime algorithm software (Edgar et al., 2011). The sequences were clustered into operational taxonomic units (OTUs) using QIIME’s pick_open_reference_otus.py with a 97% similarity cut-off. Taxonomic information was obtained using the Ribosomal Database Project classifier at a confidence threshold of 0.80 (Cole et al., 2004). In total, 936,467 effective sequences were retained after sequence information from 18 soil samples was optimized. The reads were rarefied at a 28,795-sequencing depth for each sample.

### Co-occurrence network analyses and core module identification

Microbial network analysis was used to identify clusters of microbial taxa that were highly correlated. The top OTUs, accounting for more than 80% of the relative abundance in the total community, were chosen. All pair-wise Spearman correlations between OTUs were calculated using the “Hmsic” package (Harell and Dupont, 2015) in R. To focus on OTUs that strongly co-occurred and were more likely to interact, correlations with a Spearman’s coefficient<0.8 and a P-value >0.01 were removed. The main modules in the network were visualized using Gephi (https://gephi.org/). The relative abundance of each module was calculated by averaging the standardized relative abundances (z-score) of the species to which it belonged. Modules in which taxa correlated positively with the abundance of soil functioning were also identified (Fan et al., 2021). The top 20 nodes with high degree, high closeness centrality, and low betweenness centrality scores were statistically identified as the keystone taxa (Banerjee et al., 2021; Zheng et al., 2021).

### Whole bacterial genome downloading and annotation

We selected 494 bacterial genomes from the JGI-IMG database that were previously retrieved from agricultural soils (including rhizosphere and rhizoplane samples) and retrieved their sequences from the NCBI genome database (ftp://ftp.ncbi.nlm.nih.gov/genomes/all/). Finally, a total of 2209 16S-rRNA sequences were extracted from 494 genomes (file format: _rna_from_genomic.fna.gz) using “TBtools” software (Chen et al., 2020). Online BLAST (Nucleotide BLAST: Align two or more sequences using BLAST (nih.gov)) was used to compare the representative sequences of chosen dominant bacterial OTUs (with >97% identity across the sequenced regions of the 16S rRNA genes). In total, there were 112 genomes (**Table S3**) with small subunit (ssu) rRNA 16S marker genes that could be aligned and built into a phylogenetic tree using MEGA-X and iTOL, within which, 41, 53, and 18 genomes were clustered with the dominant OTUs from Modules #1–3, respectively. The Prokka pipeline was used for gene prediction and annotation; and genes associated with carbon cycling (e.g., *abfA*, *accA*, *acsA*, *acsE*), and genes associated with nitrogen cycling (e.g., *anfG*, *hao*, *hdhA*, *napA*) were used as reference to assess potential functions (Fan et al., 2021).

### Statistical analyses

The alpha diversity was calculated using the diversity function in the “vegan” R package (Oksanen et al., 2020). Non-metric multidimensional scaling (NMDS) analysis and permutational multivariate analysis (ADONIS) were conducted using the “vegan” package (Oksanen et al., 2020). These used current literature on cultured strains to map prokaryotic clades (e.g., genera or species) to establish metabolic or other ecologically relevant functions (Louca et al., 2016). The hierarchy from phyla to OTUs was illustrated using hierarchical (i.e., tree topology) taxonomic networks in Cytoscape (version: 3.8.0) with manual adjustment (https://apps.cytoscape.org/apps/yfileslayoutalgorithms). Pairwise Spearman correlations between diversity and soil functional potentials [normalized abundance of tested functional genes, carbon-fixation potential, PNR, and DEA (z-score)] were calculated. A random forest analysis was used to estimate the importance of soil properties to explain the carbon-fixation potential, PNR, and DEA rates using the “rfPermute” R package (Eric Archer, 2021). A one-way analysis of variance (ANOVA) with Tukey’s test was conducted for multi-comparisons using R software (version 4.1.2-win).

## Results

### Changes in soil properties across irrigation practices

Significantly higher (*p*<0.05) soil moisture, ${\text{NO}}_{3}^{-}$ -N, and ${\text{NH}}_{4}^{+}$ -N were observed in the rhizosphere soil of the DSM or DDM treatment groups than in the FSM group (**Table S4**). Higher moisture was also associated with lower salinity and pH in the rhizosphere soil of the DSM or DDM groups. Drip irrigation promoted aboveground biomass, however, the limited moist area promoted shallow root distribution (**Figure S1**), resulting in lower root biomass (**Table S5**). A significantly lower (*p*<0.05) soluble sugar content in the roots and a lower WEOC in the rhizosphere soil were observed from drip irrigation practices (**Tables S4** and **S5**), suggesting that low rhizodeposition might exist under these two treatments.

### Rhizosphere bacterial communities and functional potentials across irrigation practices

Bacterial diversity was significantly lower (*p*<0.05) in the FSM than in the DDM treatment groups (**Table S6**). Indeed, permutational multivariate analysis (ADONIS) confirmed that the rhizosphere communities differed significantly in response to flooding irrigation versus drip irrigation (**Figure S2**; **Table S7**).

**Figure 1:**
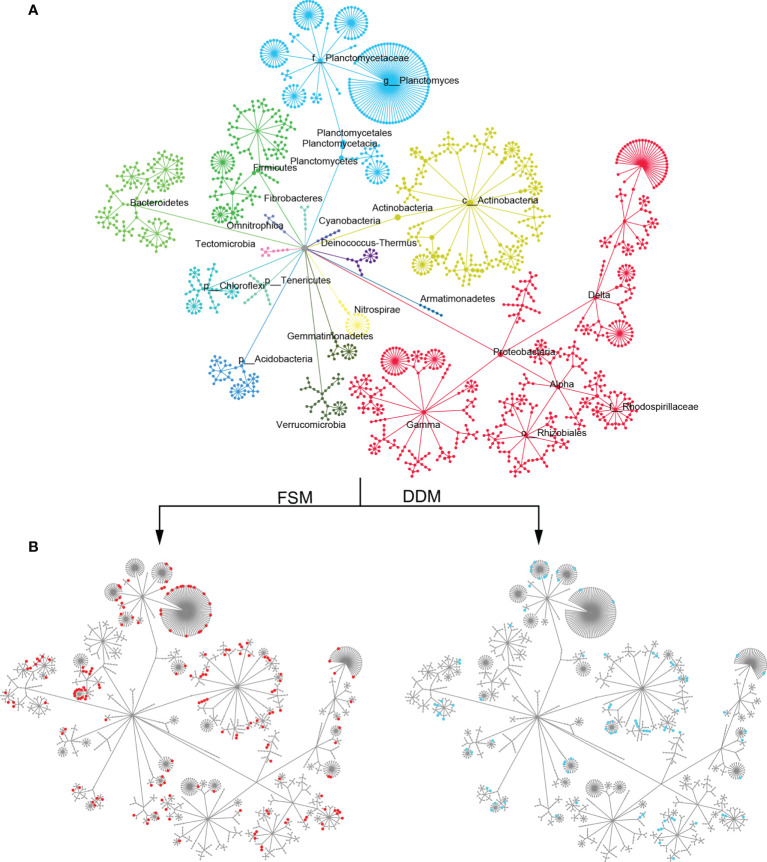
**(A)** A hierarchical taxonomic tree of bacterial communities showing OTU distribution across the different phyla. Nodes correspond to OTUs and edges represent the taxonomic path from the phylum to the OTU level. OTUs were placed at the level with the lowest possible assignment. Clusters are color-coded by their phylum-level assignment and are labeled with the phylum name. **(B)** A taxonomic tree with the same topology as shown in panel A, but with node sizes that correspond to the positive relative change (z-transformed) in abundance under flooding irrigation with film mulch (FSM) or drip irrigation with double film mulch (DDM) stands. Node colors correspond to the level of significance from highly significant (red/blue) to not significant (gray). A soft threshold using a color gradient rather than a hard cutoff was used to denote the level of significance. Nodes with *p ≤*0.05 are completely red/blue.

**Figure 2:**
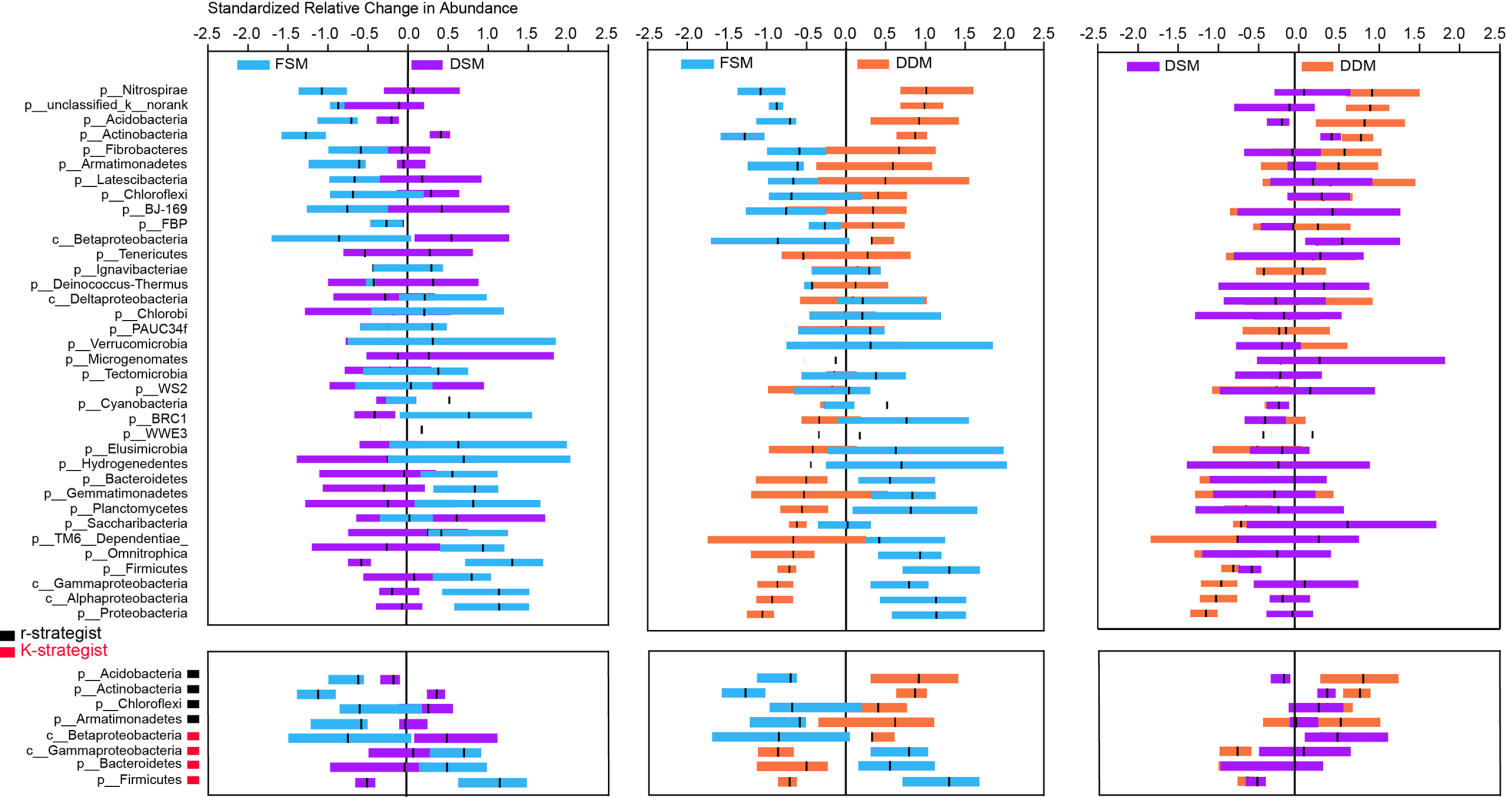
Relative changes (z-transformed) in the abundance of bacterial phyla (upper panel) as well as the major K-strategist and r-strategist groups (lower panel). K-strategist groups: *Acidobacteria*, *Actinobacteria*, *Chloroflexi*; r-strategist groups: *Betaproteobacteria*, *Gammaproteobacteria*, *Bacteroidetes*, *Firmicutes*.

Taxa responsible for the observed shifts in β-diversity were further explored at various taxonomic levels from phylum to OTU. Practice-sensitive OTUs were broadly distributed across the taxonomic hierarchy with phyla such as *Planctomyces*, *Actinobacteria*, *Bacteroidetes*, and *Gemmatimonadetes* exhibiting a clear dispersal of sensitive OTUs (**Figure 1**). Qualitatively, *Bacteroidetes*, *Omnitrophica*, *Planctomyces*, *Gemmatimonadetes*, and *Gemmatimonadetes* had fewer OTUs in the DDM than in the FSM treatment group (**Figure 1**). Consistent phylum-level differences in the bacterial community were observed among the three irrigation practices (**Figure 2**). For example, the phyla *Actinobacteria*, *Acidobacteria*, and *Armatimonadetes* showed strong increases in relative abundance under drip irrigation while *Bacteroidetes*, *Gemmatimonadetes*, and *Firmicutes* were more abundant in the rhizosphere soil that received flooding irrigation.

The abundance of the functional genes, *β-glu*, *cbbL nifH*, AOB-*amoA*, and *cnorB* was significantly lower under drip irrigation than under flooding irrigation (**Figure S3**). Meanwhile, lower RubisCO activity, nitrification, and denitrification were observed in response to drip irrigation (**Figure S3**). The process (i.e., RubisCO activity, PNR, and DEA) was correlated with soil WEOC, SOC, total N, and ${\text{NO}}_{3}^{-}$ -N levels (**Figure S4**).

### Network modules involved in soil functions

**Figure 3:**
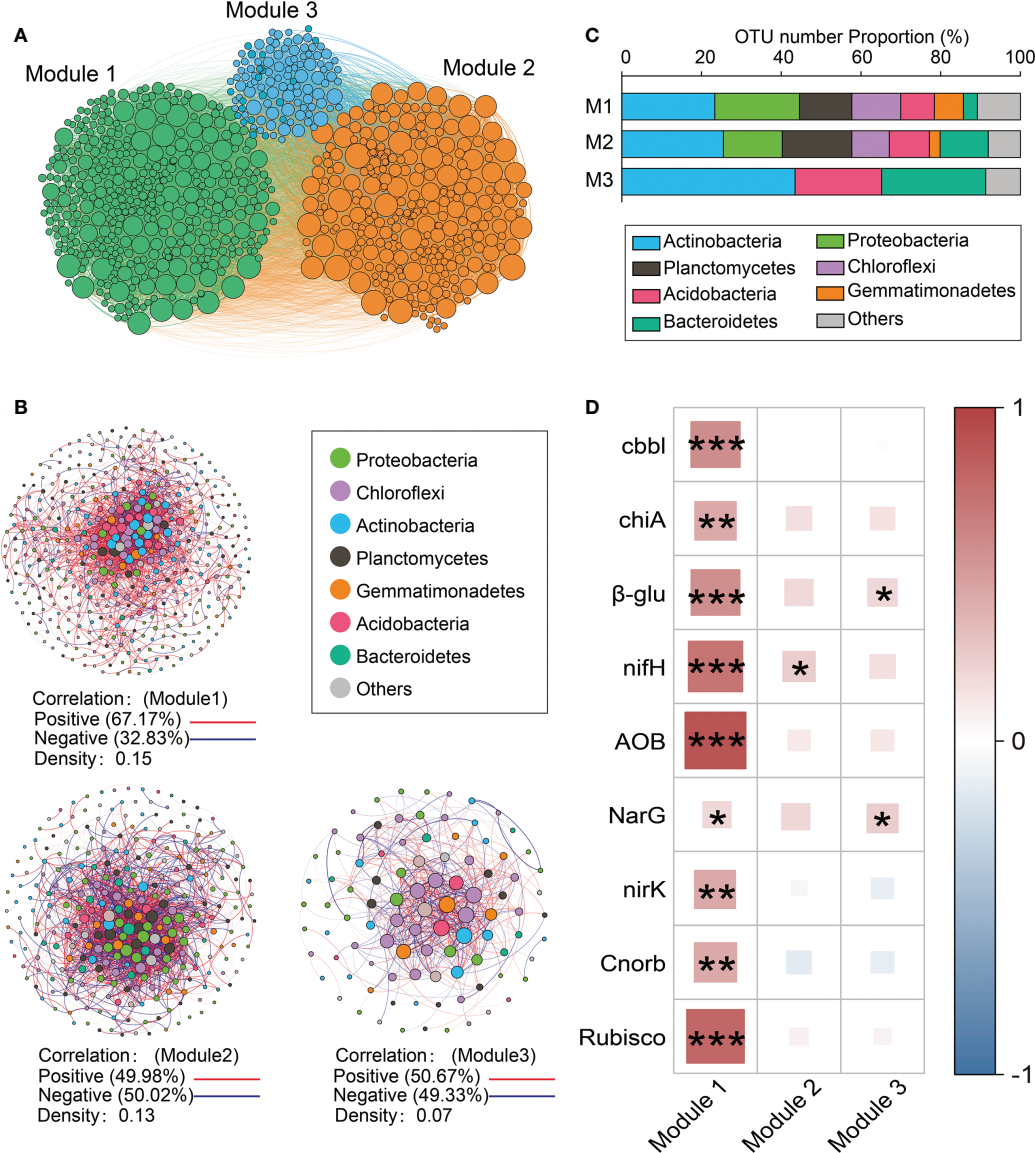
Modules based on networks. **(A)** A Network diagram with nodes colored according to each of the three main modules (Modules #1–3); **(B)** Operational taxonomic unit (OTU) number properties of the dominant taxa in the main modules; **(C)** Dominant taxa (relative abundance >0.05) from Modules #1–3, respectively. A connection stands for a strong (Spearman’s *r >*0.8) and significant (*P*<0.001) correlation. The co-occurrence network is colored by phylum. The size of each node is proportional to the relative abundance of each taxa. A red edge indicates a positive correlation between two individual nodes, while a blue edge indicates a negative correlation; **(D)** Spearman correlations between the abundance of functional genes and the relative abundance of dominant taxa from Modules #1–3 (z-score). *P < 0.05, **P < 0.01, ***P < 0.001.

Three main modules (Modules #1–3) were identified in the network (**Figure 3A**). The relative abundance of taxa belonging to Module #1 was significantly and positively correlated with the abundance of functional genes related to N and C cycling (**Figure 3D**). Given its functional importance, Module #1 will be referred to as the core module. The core module was dominated by *Actinobacteria, Proteobacteria, Chloroflexi, Planctomycetes*, and *Firmicutes* (**Figures 3B, C**), among which there were many positive correlations (density = 0.15) (**Figure 3B**). The relative abundance of the core module was lower in response to drip irrigation (**Figure S5**). The relative abundance of dominant taxa in Modules #2 and #3 showed fewer direct correlations with the abundance of functional genes than was found in the core module (**Figure 3D**). The associations between other modules and functional potential are shown in **Figure S6**. Of the 20 keystone species, 13 were in the core module (**Table S8**). They primarily belonged to *Chloroflexi* (*Caldilineaceae* and unclassified *Chloroflexi*), *Actinobacteria* (*Blastocatellaceae* subgroup 4 and unclassified *Actinobacteria*), and *Gemmatimonadetes* (*Gemmatimonadaceae*). Other members belonged to *Planctomycetes*, *Nitrospirae*, and *Proteobacteria*. Keystone taxa in Module #2 included members of *Myxococcales*, *Cytophagales*, and *Rhizobiales* (**Table S8**).

### Relationships between key taxa and soil functions

**Figure 4:**
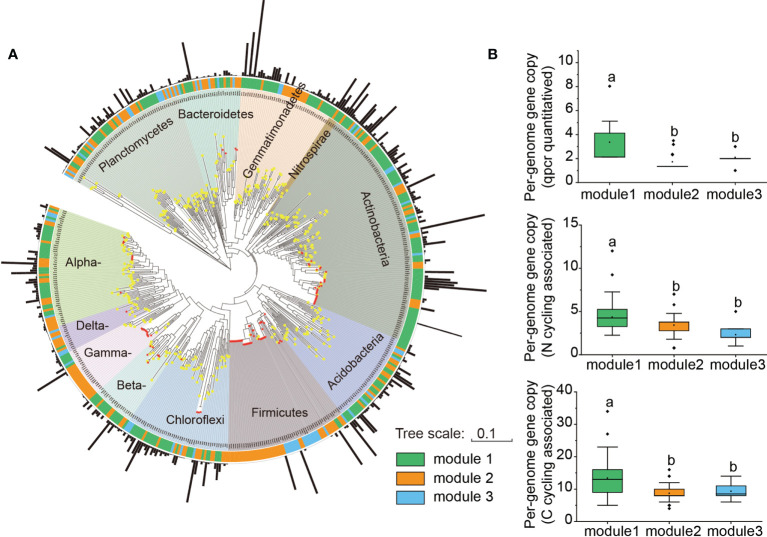
Phylogenetic properties and per-genome gene copies of mapped taxa from different modules. **(A)** Phylogenetic tree of dominant OTUs from Modules, and 35 whole genomes (with >97% identity across the sequenced region of the 16S rRNA gene) downloaded from the JGI-IMG websites (https://img.jgi.doe.gov/). Black bar: relative abundance of each OTU. Red star on the tree: ssu rRNA 16S marker genes of the whole genomes. **(B)** The normalized per-genome copy numbers of eight tested genes, and other reference genes associated with carbon (18) and nitrogen (14). The normalized copy numbers are shown as the percentage (%) of genes per genome. The diamond symbol in each box plot represents the mean value.

**Figure 5:**
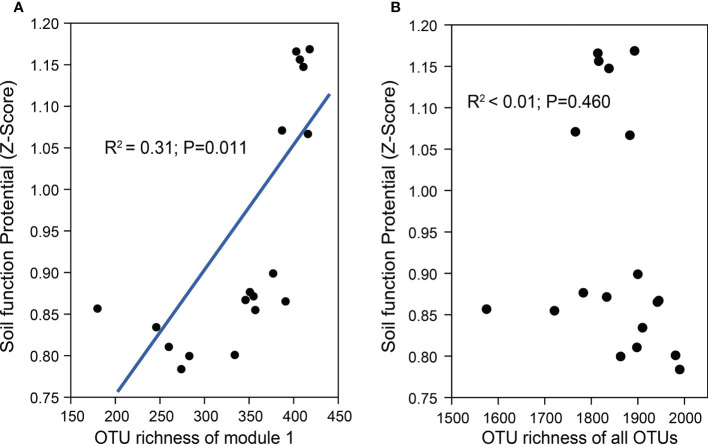
The regression relationships between the richness of the core module (Module #1) **(A)**, the richness of the whole community **(B)**, and the soil functional potentials [normalized abundance of test functional genes, RubisCO activity, PNR, and DEA (z-score)].

Further phylogenetic analysis showed that the key taxa (major taxa in Module #1) were more often phylogenetically clustered within specific bacterial taxa (e.g., *Actinobacteria*: *Actinomycetia*; *Planctomycetes*: *Planctomycetaceae*; Gemmatimonadetes: *Gemmatimonadaceae*; Alpha*-proteobacteria*: *Rhodobacteraceae*, Beta*-proteobacteria: Nitrosomonadaceae*) than the dominant taxa in other modules (**Figure 4**). The normalized per genome copy numbers of functional genes were significantly higher in the genomes clustered from key taxa than those clustered within the other two modules (**Figure 4**). In addition, the richness of the core module had a significantly positive effect on the functional potentials (**Figure 5**). We used additional examples to illustrate the link between genetic function and taxa in the core module (**Figure S7**). Specifically, the abundance of the *cbbL* and *nifH* genes correlated significantly with the richness of Alpha- and Gamma-*proteobacteria* and the richness of key taxa including *Verrucomicrobia*, *Planctomycetes* and *Gemmatimonadetes* correlated significantly with the abundance of *β-glu*, AOB-*amoA*, and *cnorB*.

## Discussion

### Distinct responses of bacterial communities to irrigation practices

There were only subtle differences in bacterial α-diversity between the two drip irrigation practices. However, the Shannon indices of both drip irrigation practices were significantly higher than the index associated with flooding irrigation (**Table S6**), suggesting that drip irrigation is associated with increased rhizosphere bacterial diversity. This is not surprising given that lower salinity and high moisture are suitable for bacterial growth. At the phylum level, *Bacteroidetes*, *Firmicutes*, and Gamma*-proteobacteria* were more abundant in response to flooding irrigation, while *Actinobacteria*, *Acidobacteria*, and *Armatimonadetes* were more abundant under drip irrigation (**Figure 1**). The K - and - r selection theory (Fierer et al., 2007) may partially explain shifts in taxonomic groups. For example, *Actinobacteria* play a vital role in the soil C-cycle by degrading recalcitrant carbon and are highly resistant to C starvation (Rosenberg et al., 2014; Hartmann et al., 2017). *Acidobacteria* and *Armatimonadetes* are also able to metabolize complex carbohydrates (Kielak et al., 2016; Hartmann et al., 2017). These K-strategist groups often dominate in soils with low carbon availability (Ali et al., 2018; Razanamalala et al., 2018). In contrast, fast-growing r-strategist organisms such as *Gamma-proteobacteria*, *Bacteroidetes* and *Firmicutes* predominate in soils with highly labile organic substrates (Fierer et al., 2007; Fierer et al., 2012; Li et al., 2019). Taken together, it is likely that decreased carbon availability (e.g., SOC, WEOC) under drip irrigation allowed K-strategists to be outcompeted in the rhizosphere. However, it was notable that Beta*-proteobacteria*, which is considered an r-strategist (Fierer et al., 2007), was highly abundant in response to drip irrigation (**Figure 2**). Thus, an assessment of the effects of different irrigation treatments on the bacterial activity at different levels is necessary for a more complete understanding of the environment.

### Core module association with soil functions

The richness of bacterial taxa in the core module (Module #1), including Alpha-, Beta- and Gamma-*proteobacteria*, *Ardenticatenia*, *Verrucomicrobia*, *Planctomycetes*, and *Gemmatimonadetes* was positively associated with the abundance of functional genes (**Figure 3**). Alpha- and Gamma-*proteobacteria* were the common autotrophic bacterial classes (Xiao et al., 2018). Some taxa from these two classes that were included in the current study, *Paracoccus* and *Dokdonella*, are important contributors to microbial CO_2_ assimilation (Zhou et al., 2019), while other taxa, including *Azospirillum*, *Bradyrhizobium*, and *Rhodospirillaceae* are N fixers (Nelson et al., 2016). Moreover, the taxa of *Nitrosomonadaceae* (*Nitrosomonadaceae*) from the core module, are shown to contain ammonia oxidizers (Prosser et al., 2014). *Ardenticatenia* belongs to *Chloroflexi* and members of this phylum are positively correlated with simple carbohydrate metabolic pathways (Pérez Castro et al., 2019). *Verrucomicrobia*, *Planctomycetes*, and *Gemmatimonadetes* are significantly positively correlated with the abundance of functional genes including those involved in C fixation, C degradation, and N cycling (Fierer et al., 2013; Shi et al., 2020). Phyla *Gemmatimonadetes* and *Verrucomicrobia* are also considered as N cycle-related bacteria and are enriched in healthy plant rhizosphere (Ge et al., 2021). Taxa from the core module also had higher normalized per-genome copy numbers of functional genes associated with C and N cycling (**Figure 4**), suggesting that this module had greater nutrient cycling potential.

It is important that most of the keystone taxa in this study were associated with the core module (**Table S8**). The disappearance of keystone taxa may cause disruptions in network modules (Guimerà and Nunes Amaral, 2005), suggesting that the core module is critical for maintaining network structure and soil functioning in the rhizosphere. Other keystone taxa, including those from *Rhizobiales*, *Myxococcales*, and *Cytophagaceae*, exist in Module #2. *Rhizobiales* members are found as keystone taxa across different ecosystems (Banerjee et al., 2018). *Myxococcales* and *Cytophagaceae* (*Cytophagales*) are groups known for their predatory lifestyle (Emmett et al., 2021), which may explain why Module #2 had the highest negative correlation.

### Drip irrigation practices reduced core module richness and soil functioning

Drip irrigation also reduced the rhizosphere soil functional potential by affecting the richness of the core module (**Figure 5**). Many variations caused by drip irrigation were able to inhibit microbial extracellular enzyme and functional gene expression. For example, two labile carbon hydrolase-coding genes, *β-glu* and *chi-A*, decreased by different amounts, suggesting that drip irrigation may reduce the production of the corresponding extracellular enzymes, and lower the efficiency of labile carbon decomposition. This result may be explained by the reduced richness of the *Verrucomicrobia*, *Planctomycetes*, and *Gemmatimonadetes* taxa. In addition, *cbbL*-carrying obligate autotrophs, not facultative autotrophs, are strongly and positively correlated with RubisCO activity (Yuan et al., 2012a; Yuan et al., 2012b; Wu et al., 2015; Lynn et al., 2017). Available N (e.g., ${\text{NO}}_{3}^{-}$ -N) decreases soil RubisCO activity by reducing obligate autotrophs (Zhou et al., 2019). Thus, the accumulation of ${\text{NO}}_{3}^{-}$ -N under drip irrigation may decrease soil obligate autotroph abundance. According to previous studies, N fertilizes against N fixation and specific groups of N fixers (Fan et al., 2019) and high ${\text{NO}}_{3}^{-}$ is negatively correlated with the abundance of denitrifying microbes (Shen et al., 2010). In addition, soil carbon is critical for the activity of denitrifiers, and denitrification is usually found in soils with both a high TOC and moisture content (Enwall et al., 2010; Banerjee and Siciliano, 2012; Schulz et al., 2017). In the current study, drip irrigation enriched ${\text{NO}}_{3}^{-}$ -N but decreased SOC and WEOC, which could be because fewer key taxa were involved in N fixation or nitrification and denitrification.

**Figure 6:**
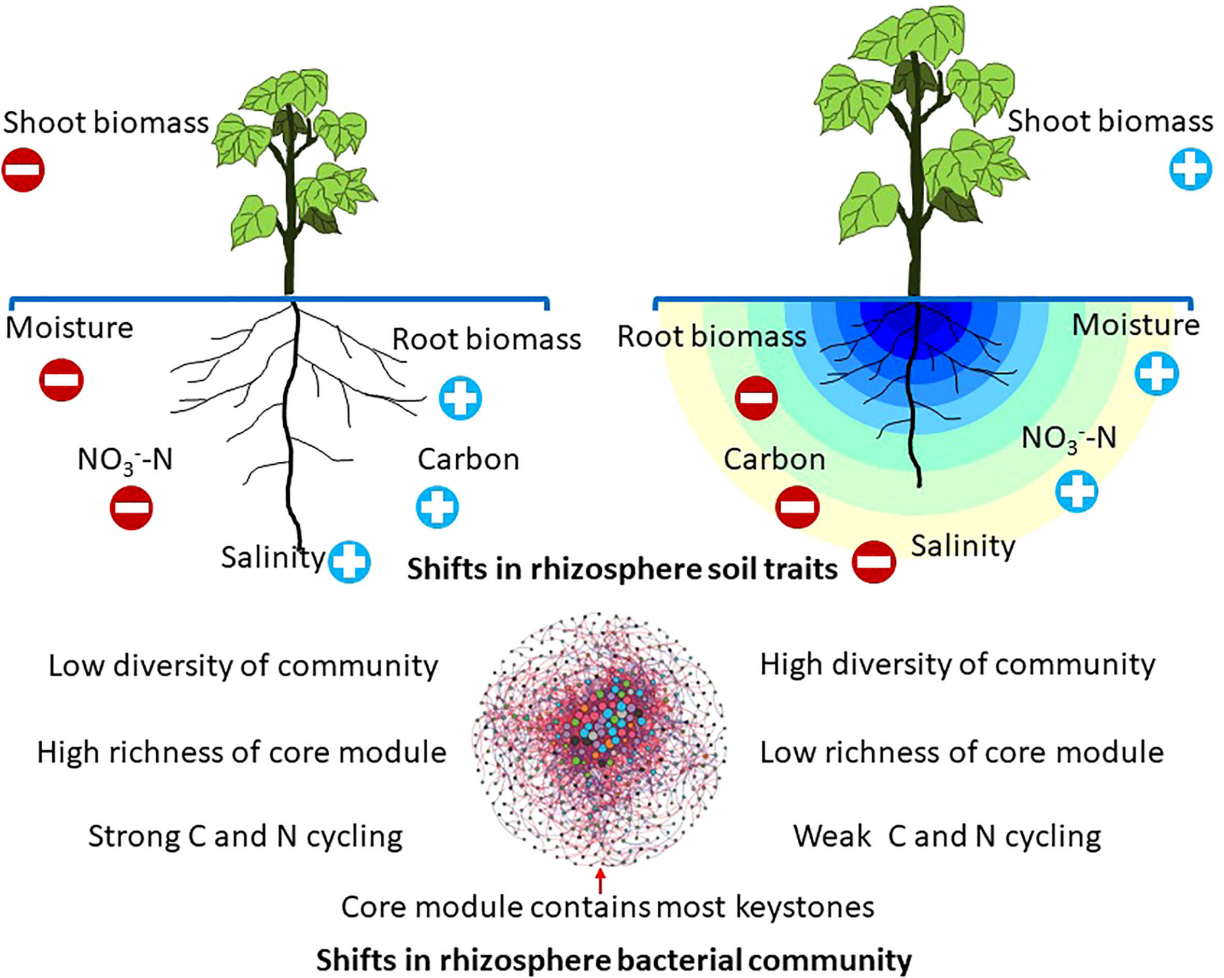
A theoretical framework exploring the effects of different practices on bacteria (structure, functional potential) in relation to multiple factors. Practices affect plant growth and rhizosphere soils, which in turn influence the rhizosphere bacterial community. The richness of the core module can be altered by agricultural practice. The pivotal changes in the bacteria-driven nutrient process are presented.

In summary, this study built a theoretical framework to characterize the effects of drip irrigation with film mulch on the rhizosphere bacterial communities (**Figure 6**). Results showed that rhizosphere bacteria, which benefit from reduced salinity and moisture, may promote community diversity, while limited carbon substrate availability caused many K-strategist groups to occupy the communities. Furthermore, WEOC, SOC, and ${\text{NO}}_{3}^{-}$ -N induced under drip irrigation may reduce the richness of the core module, which could suppress the biogeochemical cycling of C and N. It should be noted, however, that plant and soil sampling was only conducted during one growth stage in this study. Rhizosphere fungal communities can be impacted by the interactions between tillage practices and growth stage (Li et al., 2021). In addition, beneficial and tolerant microbes can be stimulated by certain long-term agricultural management practices (Fan et al., 2021). Thus, it will be necessary to evaluate the temporal consistency of these findings.

## Conclusions

This study revealed a strong effect of drip irrigation on the rhizosphere bacterial community of cotton. While this practice enhanced bacterial diversity, changes in the quantity and/or quality of plant-derived inputs, such as root biomass and carbon, and ${\text{NO}}_{3}^{-}$ -N concentration may suppress the richness of the core module and its associated functional C and N cycling potentials. These findings contribute to our understanding of the linkage between functional potentials and the rhizosphere bacterial community under different irrigation practices.

## Data availability statement

The datasets presented in this study can be found in online repositories. The names of the repository/repositories and accession number(s) can be found below: https://www.ncbi.nlm.nih.gov/genbank/, PRJNA793144.

## Author contributions

SZ and CT: Conceptualization, Investigation. SZ and BP: Software, Writing- Original draft preparation. WM: Formal analysis. SB and SZ: Validation, Writing - Review and Editing. All authors contributed to the article and approved the submitted version.

## Funding

This work was supported by the National Natural Science Foundation of China (grant nos. 31971448 and U1803233), the Foundation of Science & Technology Department of Xinjiang Uygur Autonomous Region (No. 2019XS28), the “Western young scholars” project (Grant NO. 2019-XBQNXZ-A-006), the research programs (grant nos. 2020DB001, 131965KYSB20190083 and ZD2022D001) and the Youth Innovation Promotion Association CAS (grant no. 2020433).

## Acknowledgments

We thank the editor and reviewers for their constructive suggestions and insightful comments. We are grateful to Dr. Ke Zhang and Dr. Shoule Wang for their help in collecting samples.

## Conflict of interest

The authors declare that the research was conducted in the absence of any commercial or financial relationships that could be construed as a potential conflict of interest.

## Publisher’s note

All claims expressed in this article are solely those of the authors and do not necessarily represent those of their affiliated organizations, or those of the publisher, the editors and the reviewers. Any product that may be evaluated in this article, or claim that may be made by its manufacturer, is not guaranteed or endorsed by the publisher.
