# Supplementary material for: Contrasting effect of irrigation practices on the cotton rhizosphere microbiota and soil functionality in fields
Source: Front Plant Sci. 2022 Oct 18;13:973919. doi: 10.3389/fpls.2022.973919 (PMC9623166; doi:10.3389/fpls.2022.973919)
Supplement: Supplementary file 1 [file Image_1.pdf]

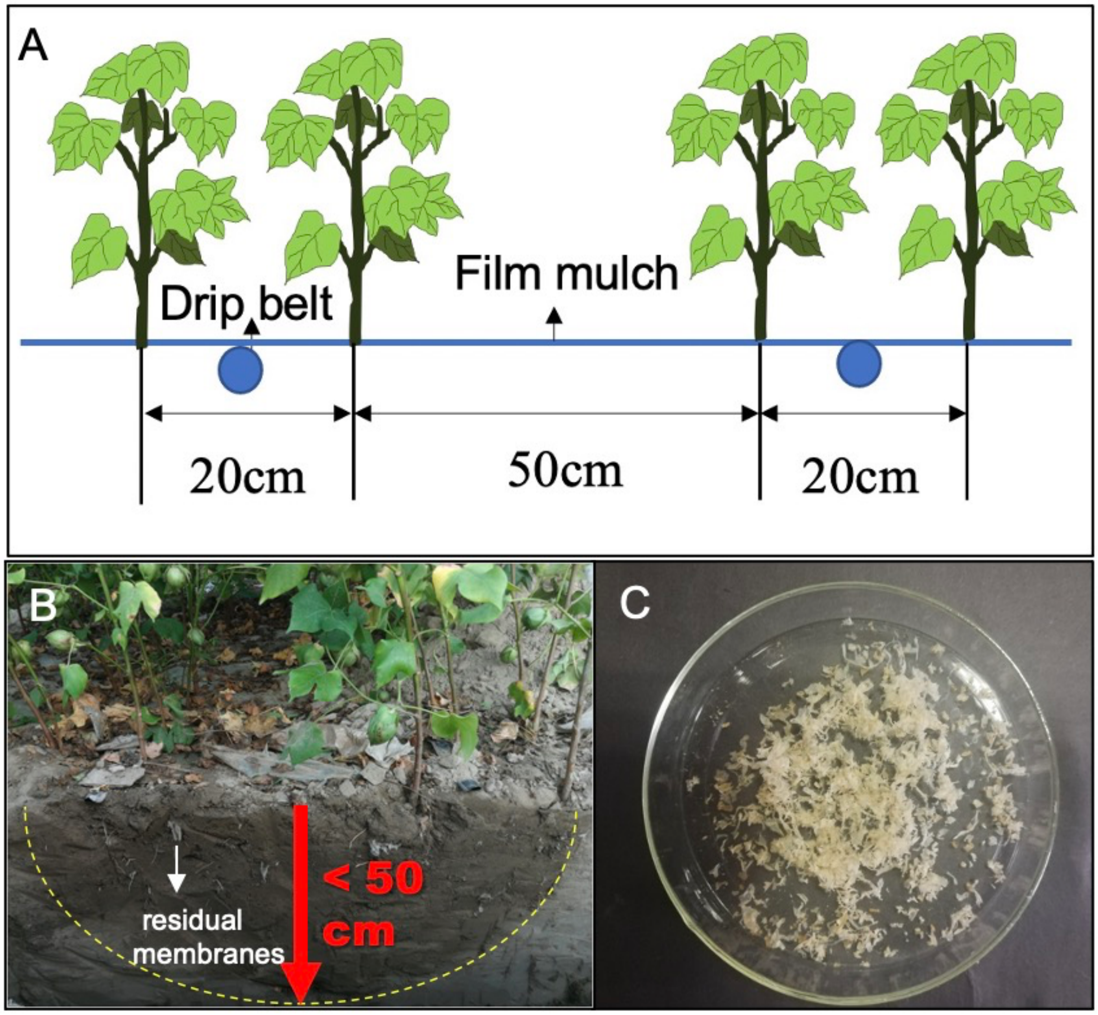

Fig. S1 A: Layout of the experimental plot. Two rows were set on both sides of the drip irrigation line. The surface of rows was covered with plastic film. B: The semidiameter of moist area was less than 50 cm in the field. C: residual membranes collected from the loose soils of roots.
