# Supplementary material for: Contrasting effect of irrigation practices on the cotton rhizosphere microbiota and soil functionality in fields
Source: Front Plant Sci. 2022 Oct 18;13:973919. doi: 10.3389/fpls.2022.973919 (PMC9623166; doi:10.3389/fpls.2022.973919)
Supplement: Supplementary file 2 [file Image_2.pdf]

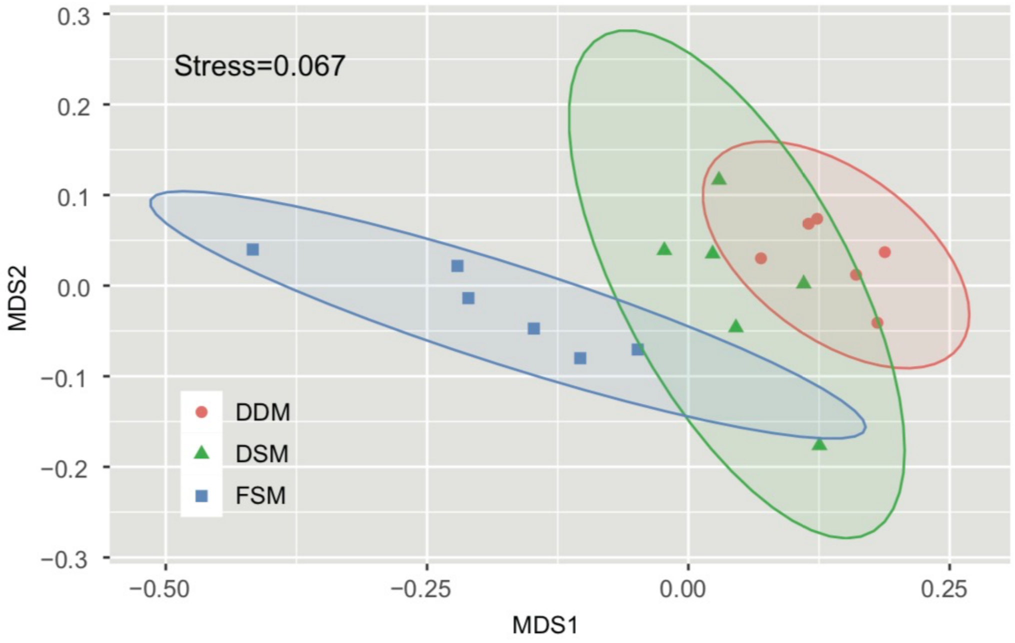

Fig. S2 Community structure of rhizosphere bacteria among three treatments. Non-metric multidimensional scaling ordination of bacterial communities based on Bray–Curtis distances. FSM: flooding irrigation under single film mulch; DSM: drip irrigation under single film mulch; DDM: drip irrigation under double film mulch
