# Supplementary material for: Contrasting effect of irrigation practices on the cotton rhizosphere microbiota and soil functionality in fields
Source: Front Plant Sci. 2022 Oct 18;13:973919. doi: 10.3389/fpls.2022.973919 (PMC9623166; doi:10.3389/fpls.2022.973919)
Supplement: Supplementary file 3 [file Image_3.pdf]

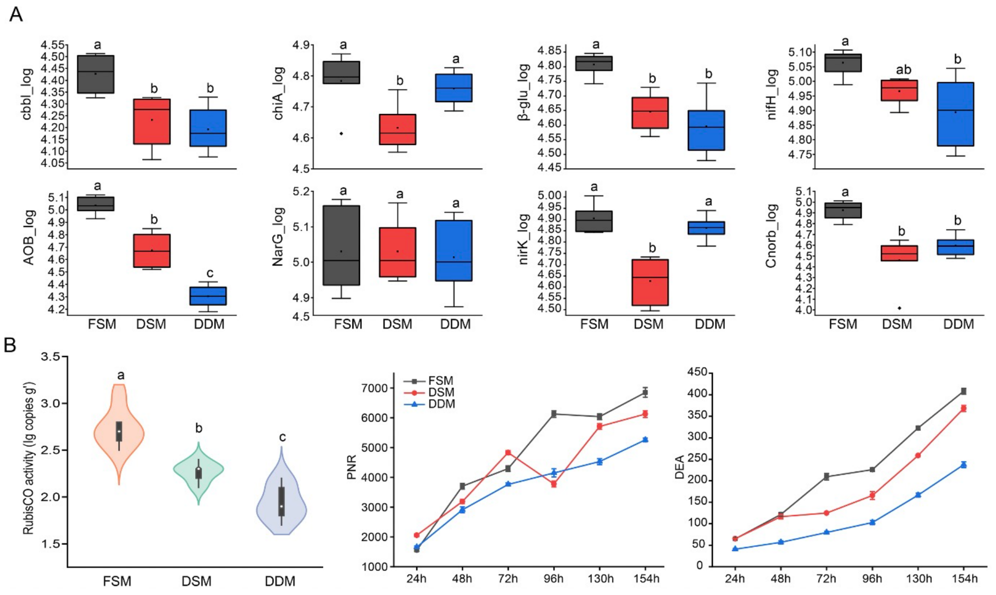

Fig. S3 A: Gene abundances involved in C or N cycling are shown as log copy numbers per gram dry soil. B: Changes in soil RubisCO activity, potential nitrification rate (PNR) and denitrification rate (DEA); Different letters above the boxes indicate significant differences at $P < 0.05$
