# Supplementary material for: Contrasting effect of irrigation practices on the cotton rhizosphere microbiota and soil functionality in fields
Source: Front Plant Sci. 2022 Oct 18;13:973919. doi: 10.3389/fpls.2022.973919 (PMC9623166; doi:10.3389/fpls.2022.973919)
Supplement: Supplementary file 4 [file Image_4.pdf]

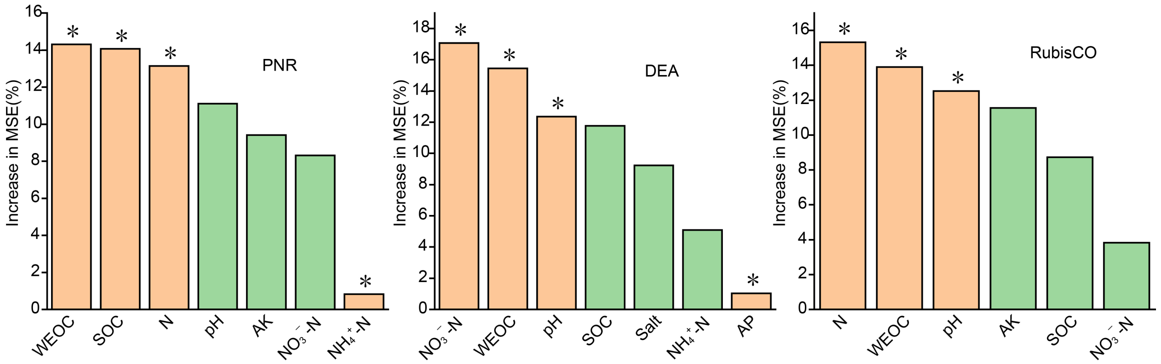

Fig. S4 We used random forest analysis to determine the predictors that best explained potential nitrification rates (PNR), overall denitrification potential (DEA) and Rubisco. Stars indicate significance of regression slopes: * $P < 0.05$, ** $P < 0.01$, *** $P < 0.001$.
