# Supplementary material for: Contrasting effect of irrigation practices on the cotton rhizosphere microbiota and soil functionality in fields
Source: Front Plant Sci. 2022 Oct 18;13:973919. doi: 10.3389/fpls.2022.973919 (PMC9623166; doi:10.3389/fpls.2022.973919)
Supplement: Supplementary file 6 [file Image_6.pdf]

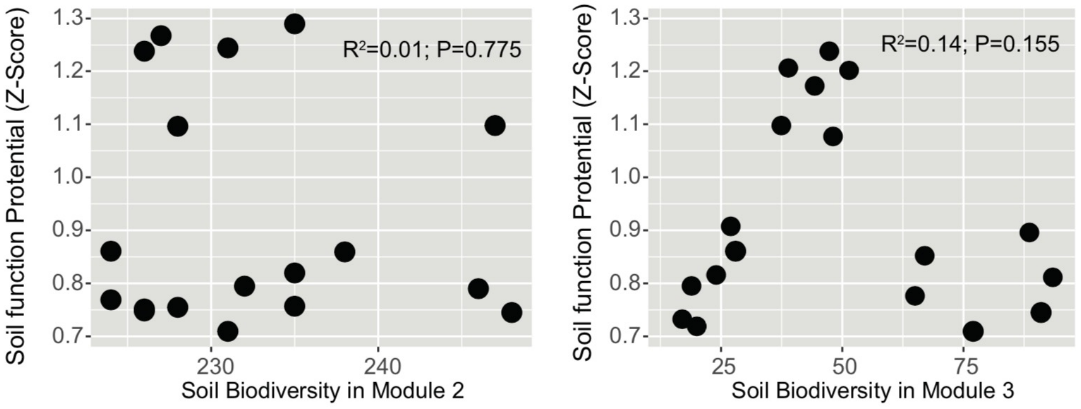

Fig. S6 Regression relationships between the biodiversity (OTU richness) of bacteria in module 2 -3 and soil functional potentials.
