# Supplementary material for: Contrasting effect of irrigation practices on the cotton rhizosphere microbiota and soil functionality in fields
Source: Front Plant Sci. 2022 Oct 18;13:973919. doi: 10.3389/fpls.2022.973919 (PMC9623166; doi:10.3389/fpls.2022.973919)
Supplement: Supplementary file 7 [file Image_7.pdf]

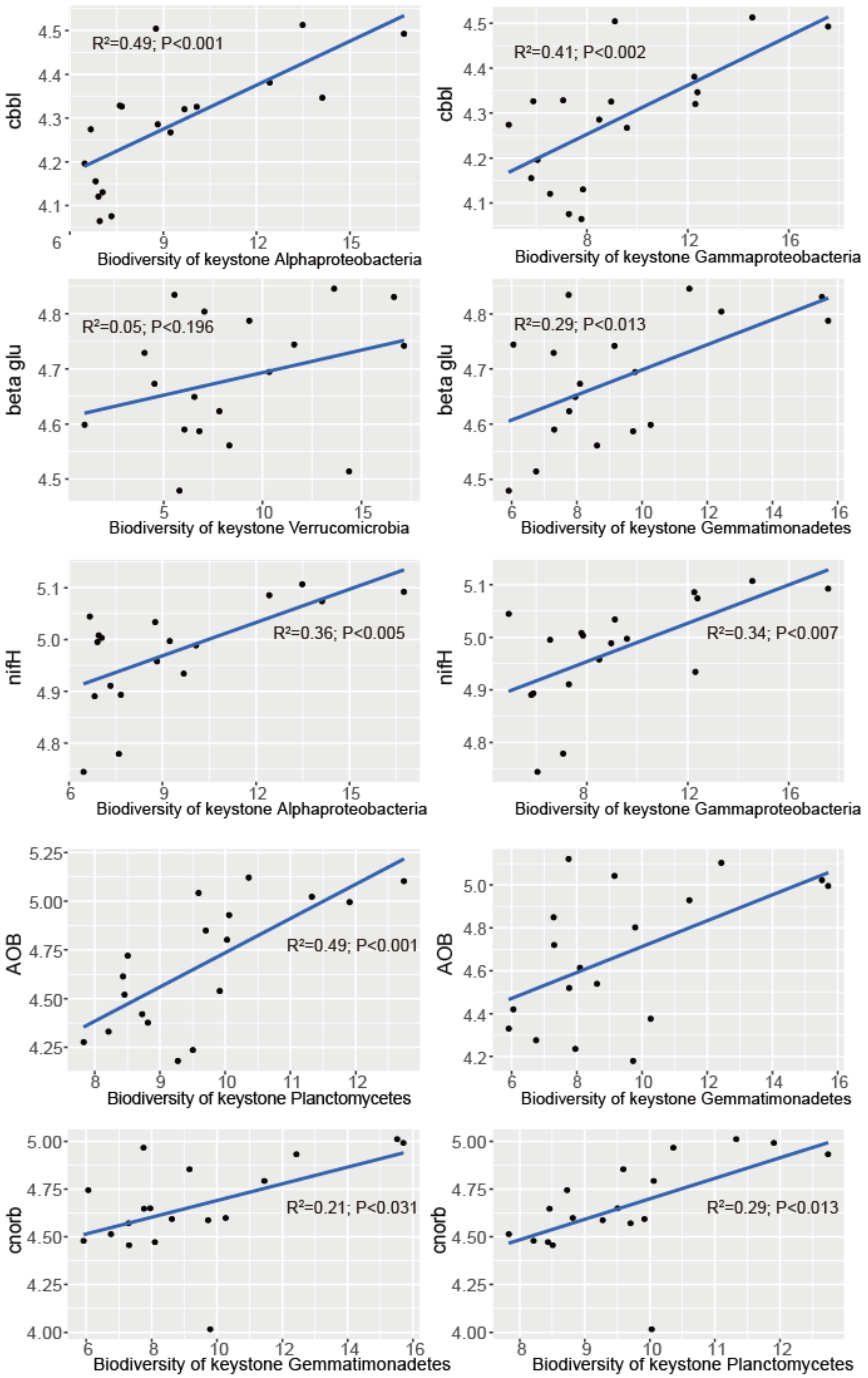

Fig. S7 Ecological relationships between biodiversity (OTU richness) of keystones and functional genes. Selected examples for the links between biodiversity of six keystone taxa in Module 1 and the abundance of five functional genes.
