# Supplementary material for: Contrasting effect of irrigation practices on the cotton rhizosphere microbiota and soil functionality in fields
Source: Front Plant Sci. 2022 Oct 18;13:973919. doi: 10.3389/fpls.2022.973919 (PMC9623166; doi:10.3389/fpls.2022.973919)
Supplement: Supplementary file 8 [file Table_1.pdf]

Table S1 Water and nitrogen fertilizer applications in the drip irrigation and flooding irrigation treatments

Treatment		Remainder	Date of application[1]									
			June			July				August		
			12 Jun<br>14 Jun	19 Jun<br>22 Jun	26 Jun<br>29 Jun	3 Jul<br>7 Jul	10 Jul<br>12 Jul	17 Jul<br>20 Jul	24 Jul<br>26 Jul	1 Aug<br>3 Aug	7 Aug<br>9Aug	15Aug<br>16 Aug
Water	Drip	4000	260	300	420	500	500	500	500	400	340	280
(m^3 ha^{-1})	Flooding	6000	–	1200	–	–	1800	–	1800	–	1200	–
Nitrogen	Drip	270	–	10	20	30	40	40	40	30	40	20
(kg N ha^{-1})	Flooding	200	–	40	–	–	–	–	100	–	60	–

[1] The upper date applies to the 2019 cropping season, and the lower date to the 2020 cropping season. The amounts of water and fertilizer applied were identical in both cropping seasons. '–' indicates no nitrogen application or no irrigation.

Urea was applied at rates of 340 for drip irrigation and 400 kg N/hm^2 for flooding irrigation in total per year. Before sowing, 20% (70 kg N/hm^2) and 50% (200 kg N/hm^2) were applied as a basal dressing for drip irrigation and flooding irrigation, respectively. The remainder was applied with irrigation. In addition, 150 kg P_2O_5/hm^2 (superphosphate) and 150 kg K/hm^2 (potassium chloride) were applied before sowing as a basal dressing.
