# Supplementary material for: Contrasting effect of irrigation practices on the cotton rhizosphere microbiota and soil functionality in fields
Source: Front Plant Sci. 2022 Oct 18;13:973919. doi: 10.3389/fpls.2022.973919 (PMC9623166; doi:10.3389/fpls.2022.973919)
Supplement: Supplementary file 9 [file Table_2.pdf]

Table S2 Real-time qPCR primers and thermal profiles used for the quantification of target genes

Gene /Primers	Target	Sequence (5' – 3')	Thermal conditions	References
chiA<br>GA1F<br>GA1R	bacterial gene coding for group A chitinases	CGTCGACATCGACTGGGARTDBCC<br>ACGCCGGTCCAGCCNCKNCCRTA	94°C, 5 min, 94°C for 60 s, 60°C for 10 s, 72°C for 60 s, 72°C for 10 min, 35 cycles	Williamson et al., 2000
β-Glu<br>βgluF2<br>βgluR4	bacterial gene coding for β-glucosidase	TTCYTBGGYRTCAACTACTA<br>CCGTTYTCGGTBAYSWAGA	95°C, 5 min, 95°C for 60 s, 53°C for 60 s, 72°C for 45 s, 72°C for 7 min, 45 cycles	Cañizares et al., 2011
cbbL<br>K2f<br>V2r	bacterial gene coding for RubisCO catalyzes	ACCACCAAGCCGAAGCTCGG<br>GCCTTCCAGCTTGCCCACCGC	95°C, 3 min, 95°C for 60 s, 62°C for 60 s, 72°C for 90 s, 72°C for 10 min, 35 cycles	Nanba et al., 2004
nifH<br>Pol-F<br>Pol-R	bacterial gene coding for nitrogenase	TGCGAYCCSAARGCBGACTC<br>ATSGCCATCATYTCRCCGGA	94°C, 30s; 58°C, 40s; 72°C, 40s, 30 cycles	Poly et al., 2001
AoB<br>amoA-1F<br>amoA-2R	bacterial gene coding for ammonia oxidase	GGGGTTTCTACTGGTGGT<br>CCCCTCKGSAAAGCCTTCTTC	95 °C for 30s, 55 °C for 60s, 72 °C for 60 s, 72 °C for 5min, 35 cycles.	Rotthauwe et al 1997
cnorB<br>cnorB2F<br>cnorB6R	bacterial gene coding for nitric oxide reductase	GGN CAY CAR GGN TAY GA<br>GAA NCC CCA NAC NCC NGC	95°C for 30 s, 55°C for 40 s, 72°C for 60 s, 35 cycles	Braker and Tiedje, 2003)
nirK	bacterial gene coding for	ATCATGGTSCTGCCGCG	95°C, 30s, 60°C for 30s, 72°C for 30 s,	Hallin et al.,

F1aCu<br>R3Cu	nitrite reductase	GCCTCGATCAGRTTGTGGTT	35 cycles	1999
narG<br>narG1960f<br>narG2650r	bacterial gene coding for nitate reductase	TAYGTSGGSCARGARAA<br>TTYTCRTACCABGTBGC	94°C, 30 s, 55°C for 30 s, 72°C for 45 s, 30 cycles	Philippot et al., 2002

References

Braker, G., Tiedje, J.M., 2003. Nitric oxide reductase (norB) genes from pure cultures and environmental samples. Applied and Environmental Microbiology. 69, 3476-3483.

Cañizares, R., Benitez, E., Ogunseitan, O.A., 2011. Molecular analyses of β-glucosidase diversity and function in soil. European Journal of Soil Biology. 47, 1-8.

Hallin, S., Lindgren, P.E., 1999. PCR detection of genes encoding nitrite reductase in denitrifying bacteria. Applied and Environmental Microbiology. 65, 1652-1657.

Nanba, K., King, G.M., Dunfield, K., 2004. Analysis of facultative lithotroph distribution and diversity on volcanic deposits by use of the large subunit of ribulose 1,5-bisphosphate carboxylase/oxygenase. Applied and Environmental Microbiology. 70, 2245-2253.

Philippot, L., Piutti, S., Martin-Laurent, F., Hallet, S., Germon, J.C., 2002. Molecular analysis of the nitrate-reducing community from unplanted and maize-planted soils. Applied and Environmental Microbiology. 68, 6121-6128.

Poly, F., Ranjard, L., Nazaret, S., Gourbiere, F., Monrozier, L.J., 2001. Comparison of nifH gene pools in soils and soil microenvironments with contrasting properties. Applied and Environmental Microbiology. 67, 2255-2262.

Rotthauwe, J.H., Witzel, K.P., Liesack, W., 1997. The ammonia monooxygenase structural gene amoA as a functional marker: Molecular fine-scale analysis of natural ammonia-oxidizing populations. Applied and Environmental Microbiology. 63, 4704-4712.

Williamson, N., Brian, P., Wellington, E.M.H., 2000. Molecular detection of bacterial and streptomycete chitinases in the environment. Antonie Van Leeuwenhoek International Journal of General and Molecular Microbiology. 78, 315-321.
