# Supplementary material for: Contrasting effect of irrigation practices on the cotton rhizosphere microbiota and soil functionality in fields
Source: Front Plant Sci. 2022 Oct 18;13:973919. doi: 10.3389/fpls.2022.973919 (PMC9623166; doi:10.3389/fpls.2022.973919)
Supplement: Supplementary file 11 [file Table_4.pdf]

Table S4 Soil properties for this study

Treatment	SOC	WEOC	TN	NO_3^--N	NH_4^+-N	AP	AK	pH	SC	SW	RM
FSM	4.35±0.06a	0.18±0.01a	0.51±0.02b	0.542±0.04c	2.22±0.1b	5.62±0.9a	284.36±18.86a	7.5±0.03a	2.36±0.13a	9.81±0.04b	27.17±1.66c
DSM	4.19±0.03a	0.15±0.01b	0.53±0.01ab	0.827±0.15b	2.38±0.19ab	4.91±0.48a	263.61±14.13a	7.36±0.03b	1.84±0.19a	12.1±0.17a	45.33±3.13b
DDM	3.54±0.1b	0.12±0.01c	0.6±0.03a	0.919±0.25a	2.76±0.11a	4.7±0.71a	282.6±19.72a	7.25±0.01c	1.18±0.12b	12.36±0.03a	74.67±2.56a

SOC: soil organic carbon (mg g^{-1}); WEOC: Water-extractable organic carbon (mg g^{-1}); TN: soil total nitrogen (mg g^{-1}); NO_3^-–N: nitrate nitrogen (mg g^{-1}); NH_4^+-N: ammonium nitrogen (mg kg^{-1}); AP: available phosphorus (mg kg^{-1}); AK: available potassium (mg kg^{-1}); SC: total water-soluble salt content (g kg^{-1}); SW: soil water content (%); RM: Residual membrane (piece m^{-2})

FSM: flooding irrigation under single film mulch; DSM: drip irrigation under single film mulch; DDM: drip irrigation under double film mulch.

The values (mean ± se) sharing the same letter are not significantly different at $P < 0.05$.
