# Supplementary material for: Contrasting effect of irrigation practices on the cotton rhizosphere microbiota and soil functionality in fields
Source: Front Plant Sci. 2022 Oct 18;13:973919. doi: 10.3389/fpls.2022.973919 (PMC9623166; doi:10.3389/fpls.2022.973919)
Supplement: Supplementary file 12 [file Table_5.pdf]

Table S5 Plant properties for this study

Treat	Shoot (g/plant)	Root (g/plant)	Soluble sugar (mg/g)	Phenol (mg/g)	Free amino acid (mg/g)
FSM	34.03 ± 1b	5.03 ± 0.13a	22.27 ± 0.36a	22.83 ± 0.56a	8.72 ± 0.2a
DSM	46.01 ± 1.23a	4.55 ± 0.18ab	18.42 ± 0.66b	23.18 ± 0.64a	8.32 ± 0.23a
DDM	48.83 ± 0.86a	4.45 ± 0.05b	17.72 ± 0.61b	22.27 ± 0.53a	8.13 ± 0.3a

FSM: flooding irrigation under single film mulch; DSM: drip irrigation under single film mulch; DDM: drip irrigation under double film mulch.

The values (mean ± se) sharing the same letter are not significantly different at $P < 0.05$.
