# Supplementary material for: Contrasting effect of irrigation practices on the cotton rhizosphere microbiota and soil functionality in fields
Source: Front Plant Sci. 2022 Oct 18;13:973919. doi: 10.3389/fpls.2022.973919 (PMC9623166; doi:10.3389/fpls.2022.973919)
Supplement: Supplementary file 13 [file Table_6.pdf]

Table S6 Diversity of soil bacterial community in different agricultural practice

	FSM	DSM	DDM
Richness	1835±19.36 b	1854±37.11 ab	1869±62.23 a
Shannon	7.87±0.07 b	8.84±0.06 ab	8.86±0.16a
Chao1	2343.88+31.38	2438.68+66.67	2402.5+102.74
ACE	2330.18+29.62	2407.49+59.04	2371.43+91.35

FSM: flooding irrigation under single film mulch; DSM: drip irrigation under single film mulch; DDM: drip irrigation under double film mulch.

The values (mean ± se) sharing the same letter are not significantly different at $P < 0.05$.
