# Supplementary material for: Contrasting effect of irrigation practices on the cotton rhizosphere microbiota and soil functionality in fields
Source: Front Plant Sci. 2022 Oct 18;13:973919. doi: 10.3389/fpls.2022.973919 (PMC9623166; doi:10.3389/fpls.2022.973919)
Supplement: Supplementary file 14 [file Table_7.pdf]

Table S7 Permutational multivariate analysis (ADONIS) of variance using Bray-Curtis distance of bacterial community under three agricultural practice

Pairs	R^2	p.value	p.adjusted
FSM vs DSM	0.319	0.006	0.009
FSM vs DDM	0.495	0.002	0.006
DSM vs DDM	0.153	0.052	0.052

FSM: flooding irrigation under single film mulch; DSM: drip irrigation under single film mulch; DDM: drip irrigation under double film mulch.
